# Supplementary material for: Content-rich biological network constructed by mining PubMed abstracts
Source: BMC Bioinformatics. 2004 Oct 8;5:147. doi: 10.1186/1471-2105-5-147 (PMC528731; doi:10.1186/1471-2105-5-147)
Supplement: Additional File 2 — The original results of the above study (non-essential files are deleted to keep the file size under the limit set by BMC bioinformatics). [file 1471-2105-5-147-S2.bz2 › chilibotAdditionalFile2/dip05/684ID9215632E17999/html/CRP_CYTR.html]

 


 **CRP** and **CYTR** 
  
Found 51 abstracts in PubMed, retrieved 05.  
 

 What does Google say? 
 PDF only 
| .edu only 

---

**Interactive relationship** (e.g. stimulation, inhibition, etc)

**Neutral relationship**- Role of protein protein bridging interactions on cooperative assembly of DNA bound  **CRP**   **CytR**   **CRP**  complex and regulation of the Escherichia coli  **CytR**  regulon.  Ref: 12667072 Biochemistry, 2003
- **CytR**  controls the balance between  **CRP**  mediated recruitment and activation of RNA polymerase and transcriptional repression.  Ref: 12667072 Biochemistry, 2003
- We have suggested that this variability affects the flexibility of  **CytR**  DNA contacts, thereby affecting the critical protein protein interactions between  **CytR**  and the cAMP receptor protein  [ **CRP** ]   **CRP**  that underlie differential repression and activation of  **CytR**  regulated genes.  Ref: 11866516 J Mol Biol, 2002
- In the deoP2 promoter of Escherichia coli, a transcription activator, cAMP  **CRP** , binds at two sites, centered at 41.5 and 93.5 from the start site of transcription, while a repressor,  **CytR** , binds to a space between the two cAMP  **CRP**  complexes.  Ref: 11574471 EMBO J, 2001
- Cooperative interactions between  **CytR** , when bound to an operator CytO located upstream of a  **CytR**  regulated promoter, and  **CRP** , when bound to flanking tandem promoters, are critical to the regulatory role of  **CytR** .  Ref: 12667072 Biochemistry, 2003
- The unlinked operons that comprise the Escherichia coli  **CytR**  regulon are controlled coordinately through interactions between two gene regulatory proteins, the cAMP receptor protein  [ **CRP** ]   **CRP**  and the cytidine repressor  **CytR** .  Ref: 12667072 Biochemistry, 2003

**Non-interactive relationship** (e.g. studied together, co-existance, homology, etc.)

- The data presented suggest that the expression of the P2 promoter, unlike that of P1, is not subjected to regulation with participation of the  **CytR**  protein and the cAMP  **CRP**  complex.  Ref: 12068544 Genetika, 2002
- Despite only weak association between  **CytR**  and  **CRP**  in solution, our results demonstrate the formation of a relatively stable complex in which the Core domain forms a protein bridge between tandem  **CRP**  dimers when bound to either udpP or deoP2.  Ref: 12667072 Biochemistry, 2003
- The mechanisms for the cAMP  **CRP**  mediated transcription activation and  **CytR**  mediated transcription repression were investigated in vitro using purified components.  Ref: 11574471 EMBO J, 2001
- However, this cytidine mediated effect varies among promoters, suggesting that coupling between cytidine binding to  **CytR**  and  **CytR**   **CRP**  association is sensitive to promoter structure.  Ref: 12667072 Biochemistry, 2003
- To investigate the chemical and structural basis for these effects, we investigated how cytidine binding affects association between  **CytR**  and  **CRP**  in solution and how it affects the binding of  **CytR**  deletion mutants lacking the DNA binding HTH domain, with tandem  **CRP**  dimers bound to either udpP or deoP2.  Ref: 12667072 Biochemistry, 2003
- After introduction of mutations affecting binding sites for  **CRP** , the negative effect of the  **CytR**  protein on promoter transcription was fully abolished.  Ref: 12391883 Genetika, 2002
- The mechanism of repression by  **CytR**  proposed in this study is that the cAMP  **CRP**  bound at 41.5 undergoes an allosteric change upon direct interaction with  **CytR**  such that it no longer maintains a productive interaction with the N terminal domain of alpha, but instead acts as a repressor to interfere with RNA polymerase acting on deoP2.  Ref: 11574471 EMBO J, 2001
- In mutants with an improved 10 region, a partial relief from the control of the cAMP  **CRP**  transcription activation complex occurred, and the negative  **CytR**  repressor regulation was reduced.  Ref: 12391883 Genetika, 2002
- These findings demonstrate that direct coupling between cytidine binding to  **CytR**  and  **CytR**   **CRP**  association does not account for promoter specific effects on cooperativity.  Ref: 12667072 Biochemistry, 2003
- In Process Citation Site directed mutagenesis was conducted in the regulatory region of the Escherichia coli udp gene at promoter sites responsible for binding regulatory proteins  **CRP**  and  **CytR**  as well as RNA polymerase.  Ref: 12391883 Genetika, 2002
